# Biogeography and eye size evolution of the ogre-faced spiders

**DOI:** 10.1038/s41598-022-22157-5

**Published:** 2022-10-22

**Authors:** Lisa Chamberland, Ingi Agnarsson, Iris L. Quayle, Tess Ruddy, James Starrett, Jason E. Bond

**Affiliations:** 1 Department of Entomology and Nematology, University of California Davis, Davis, CA 95616 USA; 2 Faculty of Life and Environmental Sciences, University of Iceland, Sturlugata 7, 102 Reykjavik, Iceland; 3 Vassar College, Poughkeepsie, NY 12604 USA

**Keywords:** Evolution, Phylogenetics, Taxonomy, Phylogeny

## Abstract

Net-casting spiders (Deinopidae) comprise a charismatic family with an enigmatic evolutionary history. There are 67 described species of deinopids, placed among three genera, *Deinopis, Menneus,* and *Asianopis,* that are distributed globally throughout the tropics and subtropics. *Deinopis* and *Asianopis*, the ogre-faced spiders, are best known for their giant light-capturing posterior median eyes (PME), whereas *Menneus* does not have enlarged PMEs. Molecular phylogenetic studies have revealed discordance between morphology and molecular data. We employed a character-rich ultra-conserved element (UCE) dataset and a taxon-rich cytochrome-oxidase I (COI) dataset to reconstruct a genus-level phylogeny of Deinopidae, aiming to investigate the group’s historical biogeography, and examine PME size evolution. Although the phylogenetic results support the monophyly of *Menneus* and the single reduction of PME size in deinopids, these data also show that *Deinopis* is not monophyletic. Consequently, we formally transfer 24 *Deinopis* species to *Asianopis*; the transfers comprise all of the African, Australian, South Pacific, and a subset of Central American and Mexican species. Following the divergence of Eastern and Western deinopids in the Cretaceous, *Deinopis/Asianopis* dispersed from Africa, through Asia and into Australia with its biogeographic history reflecting separation of Western Gondwana as well as long-distance dispersal events.

## Introduction

Once characterized as rare^1^, the net-casting spiders (family: Deinopidae; C.L. Koch, 1850) are cryptic, challenging to collect, and historically under-sampled and understudied. Living deep in understory habitats throughout tropical and subtropical regions^2^, deinopids were seldom collected^1^ and their perceived rarity is likely due to inattention and their unusual habits. Therefore, despite being charismatic spiders admired by most arachnologists, the difficulty in sampling them has resulted in very poor knowledge of the group’s evolutionary history. Deinopids, like the majority of web-spinning spiders, are sit-and-wait predators; however, they have a unique hunting strategy. Instead of sitting in their web, these spiders employ a modified orb web^3^ that they manipulate using their two anterior leg pairs. Using a silken net spun of a fuzzy, mechanical-capture silk (cribellate silk) rather than gluey silk, deinopids remain motionless until a prospective prey comes into view. They lunge into a forward strike, extending their webs with their legs and then envelope their prey, rendering them immobile^1,4–7^. They are also capable of a backward strike detecting vibrations of flying prey, hence are not entirely reliant on their enlarged eyes for prey capture^7^.

Historically, phylogenetic analyses based on morphology have divided the family into two genera: *Deinopis*, the ogre-faced spiders, aptly named for their massive posterior median eyes (PMEs), and *Menneus*, the humpback spiders^1^. Both genera share the unique net-casting hunting strategy, yet only *Deinopis* species have distinctively enlarged PMEs. Eye size, function, orientation, and visual field overlap all contribute to how spiders perceive visual signals. While some spiders have visual fields that span 360°^8^, the spider optical system is highly diversified across taxa and typically aids in prey recognition, hunting, predator avoidance, mating, and courtship^9–11^. There are two eye types, the principal eyes (typically three pairs), which capture light, and a pair of eyes (the PMEs) which are resolution-based^10,12^. In deinopids, the PMEs are forward-facing with low visual acuity and are primarily responsible for detecting motion^10^. Remarkably, *Deinopis* PMEs are the largest simple eyes of *any* arthropod and are 2000 × more sensitive to light than human photoreceptors^13^. They are a particularly important feature that enables visual hunting at night in low-light conditions^14^. Although other characters differentiate the two nominal genera, including abdominal tubercles, genitalic features, and their geographic distributions, deinopids have been notoriously difficult to diagnose based on somatic characters alone, particularly to species level^1^.

Molecular phylogenetic treatments of Deinopidae have focused on species-level relationships within *Deinopis*^15^ and a newly described genus in Asia, *Asianopis*^16^, formerly *Deinopis*. Molecular data revealed that Eastern Hemisphere *Deinopis* are more closely related to *Menneus* from South Africa than to Western Hemisphere *Deinopis*^15^. Blest et al.^17^ postulated that *Menneus* PMEs were a plausible ancestral form of the enlarged *Deinopis* PMEs. *Deinopis* paraphyly contradicts a morphology-based phylogeny of deinopids^1^, and foments questions: did deinopids gain their large PMEs within the clade, or ancestrally followed by subsequent loss, and when did this shift in PME size occur? Furthermore, did this shift in PME size occur once or multiple times across the evolutionary history of deinopids? Eye size reductions and even complete eye loss are typical in troglomorphic organisms, including crustaceans^18,19^, fish^20,21^, blind mole rats^22^, beetles^23^, and spiders^24–27^, where, in eternal darkness, energetically costly eyes may no longer offer fitness benefits^28^. Troglomorphic arthropods have been useful systems for studying the evolution and genetic underpinnings and regulation of eye size^18,29,30^; however, in arachnids, the origins, development, and evolution of eye size remains largely unexplored^12,31^. Alternatively, large PMEs could have evolved multiple times in parallel in deinopids. While energetically costly to maintain, large eye size has been associated with enhanced survival, fitness, and prey capture in vertebrates^32–35^ and invertebrates^36–38^.

**Figure 1:**
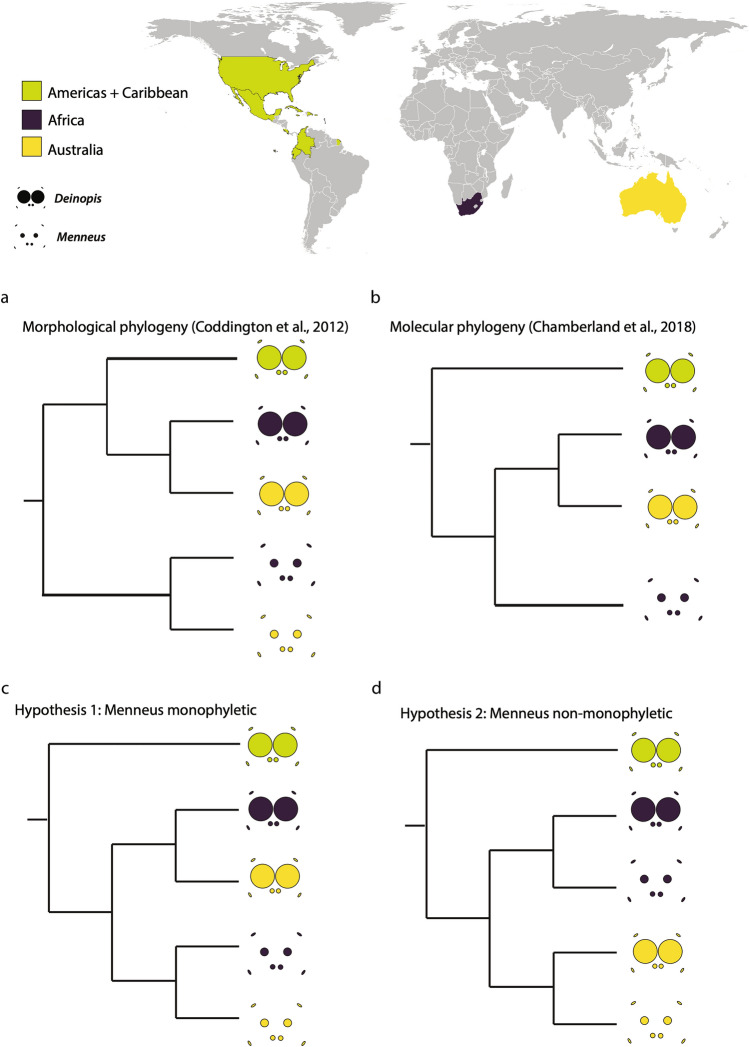
Main phylogenetic hypotheses of Deinopidae assessing the monophyly of Menneus. We included all alternative tree topologies, which include non-monophyly of *Deinopis* and *Asianopis* in Supplemental Fig. S1. Phylogenies are based on (**a**) morphological data, (**b**) incomplete molecular data. The two primary alternative hypotheses we tested with the addition of *Menneus* from Australia: (**c**) PME size is reduced once and (**d**) PME size is reduced twice, independently in Australian and South African *Deinopis.* Map was created using the base map form Wikimedia Commons (https://commons.wikimedia.org/wiki/File:BlankMap-World.svg) and country colors were modified using Adobe Illustrator (https://www.adobe.com/) (Map: Lisa Chamberland).

Trait evolution must be considered across geologic time scales in order to better understand macroevolutionary patterns. Teasing apart the role of vicariance—the geographic separation of populations over time leading to divergent species—from long-distance, often overwater, dispersal (long-distance dispersal—LDD) (e.g.^40–44^) in generating species distributions is at the center of biogeographic studies. Rigorous methods and model comparison frameworks have allowed researchers to explicitly test dispersal hypotheses^45^. Disjunct distributions of Southern Hemisphere taxa have largely been attributed to vicariance driven by the breakup of Gondwana, the southern portion of Pangaea, in the Mesozoic^46^. Deinopids were present on Gondwana prior to the breakup of the supercontinent, with the separation of Africa and South America generating the Eastern and Western Hemisphere deinopid clades^15^. Both vicariance and LDD have been important in shaping the distribution of deinopids in the Western Hemisphere^15^. Vicariance and LDD have also shaped the distributions of deinopids across the Eastern Hemisphere; however, the timing of this dispersal has not yet been extensively tested. Eastern Hemisphere deinopids contain both large-eyed *Deinopis* and *Asianopis* and small-eyed *Menneus,* and they are distributed throughout South Africa, Madagascar, Southeastern Asia, Indomalaya, and Australia. Because the Chamberland et al.^15^ molecular phylogeny of *Menneus* only contained species from South Africa, we have yet to ascertain when and where *Menneus* hypothesized eye size reduction, or *Deinopis* and *Asianopis* eye size increase, evolved. Did this evolutionary shift in eye size occur on the same continent and then the taxa dispersed (*Menneus* is monophyletic; trait conservatism) or did these shifts occur multiple times on different continents (*Menneus* is polyphyletic; trait convergence/parallel evolution) (Fig. 1)? With new *Menneus* and *Deinopis* specimens from Australia, we were able to more thoroughly test the historical biogeography of Eastern Hemisphere deinopids, while exploring the evolution of PME size across geographic time scales.

**Figure 2:**
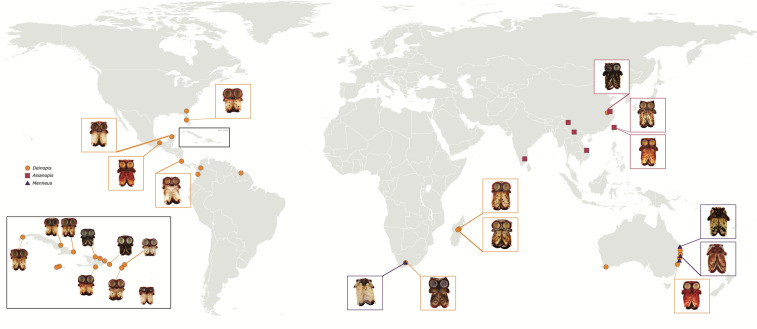
Sampling map of deinopids. Localities colored by current taxonomic genus. Map was created using the base map form Wikimedia Commons (https://commons.wikimedia.org/wiki/File:BlankMap-World.svg) Map: Lisa Chamberland).

In the study, we have integrated a taxon-rich COI dataset with a locus-rich UCE dataset to infer phylogenetic relationships within the family Deinopidae and explicitly test the monophyly of *Menneus, Asianopis,* and *Deinopis*. We expanded taxon sampling beyond Chamberland et al.^15^ to include *Menneus* specimens from South Africa and Australia and *Deinopis* from Australia, South Africa, Taiwan, Madagascar, and Mexico (Fig. 2). Specifically, we seek to infer the first genus-level phylogeny of the family and explicitly evaluate the evolution of eye reduction in *Menneus.* We tested the hypotheses that the ‘regular’ sized PMEs of *Menneus* either (1) represent an ancestral state, (2) evolved once from a common ancestor shared with *Deinopis* that then dispersed to other continents, or (3) arose independently from *Deinopis* in South Africa and Australia. Finally, we examine the ancient Gondwanan biogeographic history of Deinopidae within the context of the newly derived phylogenetic hypothesis.

## Results

### Phylogenetics

**Figure 3:**
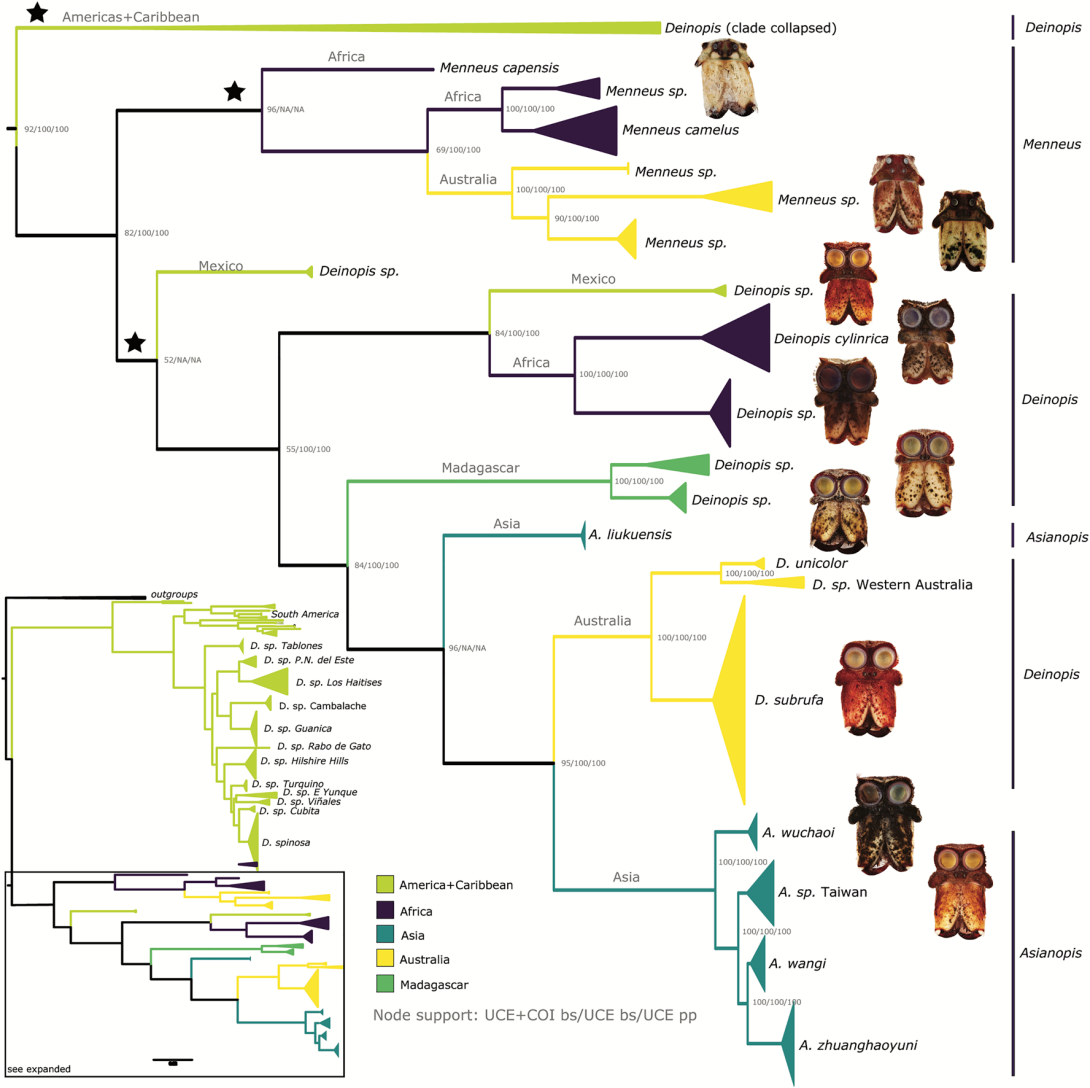
Maximum-likelihood phylogenetic tree of Deinopidae inferred using the COI + UCE (75% occupancy) matrix. Clades are colored by geographic location. The concatenated dataset resolves the uncertain placement of Australian *Menneus* and strongly supports the monophyly of *Menneus* sister to Eastern Hemisphere *Deinopis.* Numbers at nodes indicate bootstrap support values. Black stars indicate proposed genera (*Deinopis, Menneus,* and *Asianopis*).

**Figure 4:**
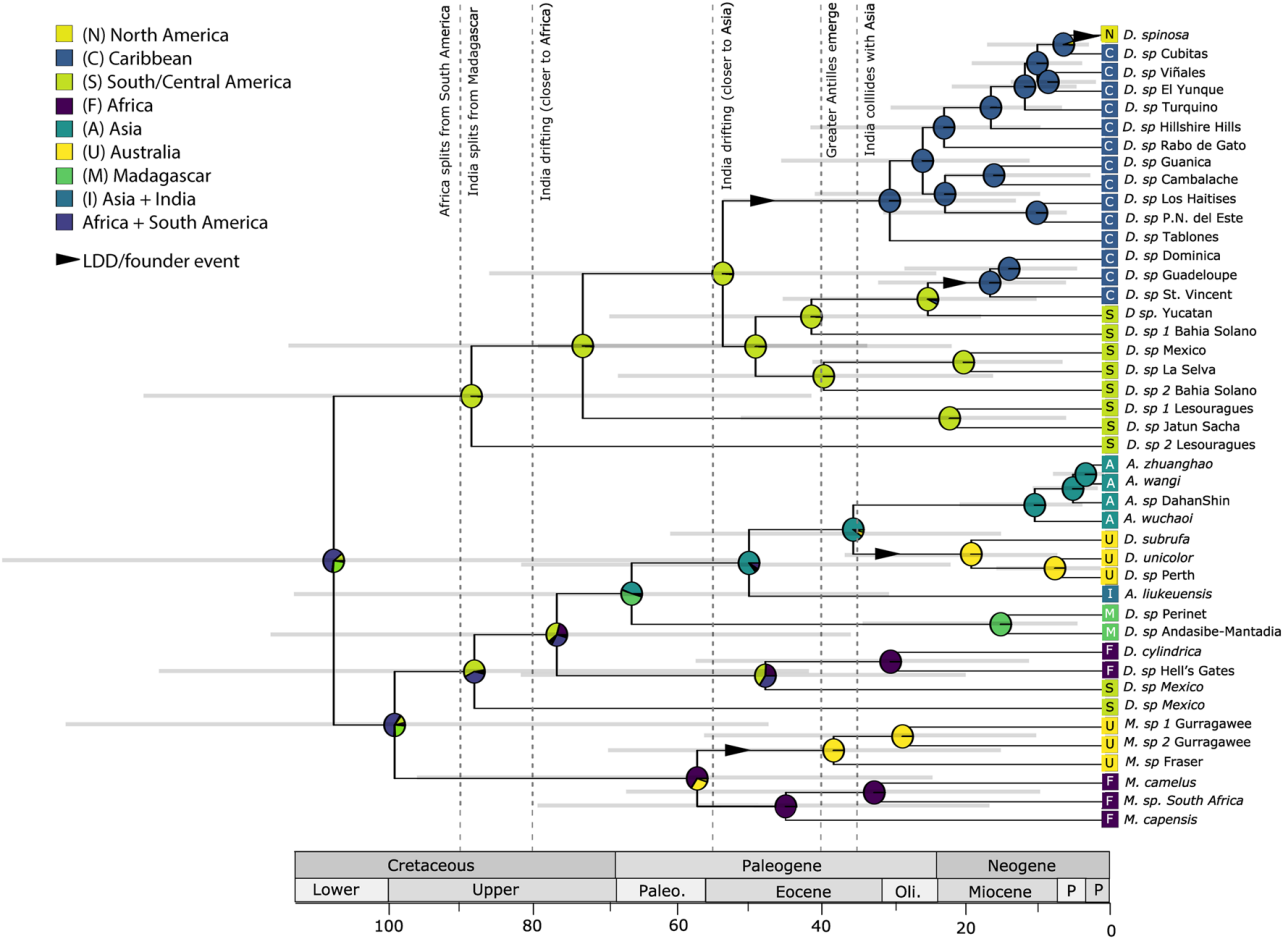
Dated phylogeny inferred using mcmcTREE. Pie charts are colored by geographic range.

The taxon-rich cytochrome-oxidase I (COI) dataset comprised 258 deinopids and 4 Uloboridae outgroup individuals. Phylogenetic analysis of the COI dataset yielded weak nodal support for a number of important clades, including the placement of *Menneus*. To resolve the ambiguous phylogenetic relationships, we generated UCEs for 40 deinopid individuals across all major clades. We found strong support for *Menneus* monophyly and *Deinopis* paraphyly with the UCE and UCE + COI concatenated datasets (Figs. 3, 4; Table 1). Although the COI-only analyses did not support the monophyly of *Menneus*, nodal support was low (Supplementary Fig. S2). Furthermore, the tree topology tests on the UCE dataset supported the unconstrained tree, with *Menneus* monophyletic and *Deinopis* and *Asianopis* both paraphyletic, and rejected all four alternative tree topology hypotheses (see “Methods”; Table 1; Supplementary Table S1).

**Table 1:** Divergence times and average marginal likelihoods and 95% HPD of deinopids based on UCE + COI concatenated MCC phylogeny.

Major divergences	Age (Ma)	lower 95% HBD	upper 95% HBD
Deinopidae diverges from Uloboridae	127.74	57.83	209.49
*Menneus* diverges from *Asianopis*	97.67	46.53	142.13
Australian *Menneus* diverges from African *Menneus*	56.42	24.22	94.35
South African *Asianopis* diverges from Malagasy + Australian + Asian *Asianopis*	75.46	35.34	114.26
Malagasy *Asianopis* diverges from Australian + Asian *Asianopis*	65.24	30.13	111.08
*A. luikenisis* (India, China, Taiwan) diverges from Australian + Asian *Asianopis*	49.17	21.76	80.17
Asian *Asianopis* diverges from Australian *Asianopis*	34.98	14.91	59.9
South American *Deinopis* diverges from Caribbean *Deinopis*	52.46	23.71	84.51
**Crown ages**			
Deinopidae	105.9	50.75	150.92
*Asianopis* (and Eastern Hemisphere *Deinopis*) clade	86.73	41.03	129.45
*Menneus*	56.42	24.22	94.35
*Deinopis* (Western Hemisphere *Deinopis*) clade	87.01	40.7	131.5
Caribbean *Deinopis* clade	30.26	12.88	52.68

We found strong support for Eastern and a Western Hemisphere deinopid clades across all phylogenetic analyses (Figs. 3, 4). The Western Hemisphere clade contained a Caribbean clade nested within a South and Central American grade, which was consistent with the phylogeny from Chamberland et al.^15^. There were nine nominal and nine putative species nested within the Eastern Hemisphere clade, including: three *Menneus* species from South Africa and three from Australia; two *Deinopis* species from Madagascar, three from South Africa, three from Australia, and at least one from Mexico; and five *Asianopis* species, including an undescribed *Asianopis* species from Taiwan (Fig. 3). The relationships of nominal genera differed between the COI only analyses and the analyses that contained UCE data; however, bootstrap support at these nodes were low and were most likely the result of using a single mitochondrial gene^47–52^. The addition of the UCE backbone to the COI dataset resulted in higher bootstrap support at the nodes. Thus, for all subsequent inferences and interpretations, we only considered the UCE only and UCE + COI phylogenies. The Eastern Hemisphere deinopid clade contained *Menneus, Asianopis,* and paraphyletic Eastern hemisphere *Deinopis* (Fig. 3). African, Australian, and Malagasy *Deinopis* were each monophyletic. We found *D. cylindrica* and an undescribed species, *D. sp* Hell’s Gates within an African clade. There were also two undescribed species within the Malagasy clade, *D. sp* Perinet and *D. sp* Andasibe-Mantadia. Finally, all phylogenetic analyses indicated three *Deinopis* clades in Australia, including *D. subrufa* in Eastern Australia*, D. unicolor* in Western Australia, and an undescribed species in Western Australia (Figs. 3, 4).

There is a well-supported relationship of a group of Mexican *Deinopis* sister to South African *Deinopis* (Fig. 3; Supplementary Figs. S3 and S4), thus supporting at least one Mexican group in the Eastern Hemisphere *Deinopis* + *Asianopis* clade. Individuals from this clade share morphological traits that characterize Eastern Hemisphere *Deinopis* and *Asianopis*, including bulky (as opposed elongated and narrow) abdomens. A second Mexican clade was sister to the entire Eastern *Deinopis* + *Asianopis* clade; however, this result was weakly supported and these individuals were represented by COI data only (Supplementary Fig. S5).

Within *Asianopis,* we recovered UCE data for *A. wangi* and *A. wuchoai. A. wangi, A. wuchaou, A. zhuanghaoyuni,* and *A. sp* from Taiwan all formed a clade sister to Australian *Deinopis. Asianopis* was paraphyletic in the UCE + COI phylogenetic inference with *A. liukuensis* sister to an Australian *Deinopis* + *Asianopis* clade. We were unable to recover UCEs for *A. liukuensis*; however, the COI only and COI + UCE concatenated phylogenetic reconstructions nested Australian *Deinopis* within the *Asianopis* clade. The *A. liukuensis* clade comprised specimens from China and India from Lin et al.^16^ and one specimen from Taiwan from the current dataset. Still, in all analyses, *Asianopis* (excluding *A. liukuensis*) was sister to Australian *Deinopis.*

Both the UCE only and COI + UCE analyses supported *Menneus* as monophyletic and sister to Eastern Hemisphere *Deinopis* + *Asianopis* clade*.* Both African and Australian *Menneus* were reciprocally monophyletic with three putative species in each geographic clade. We recovered UCEs for *M. camelus* and an undescribed species of *Menneus* from Africa, but only COI data for *M. capensis.*

### Divergence time estimates and biogeography

Eastern and Western Hemisphere deinopids diverged in the lower Cretaceous and had an ancestral range in Africa + Australia + Asia (Fig. 4, Table 1). Following a vicariant divergence of Eastern and Western deinopids, *Menneus* diverged from the rest of the Eastern hemisphere deinopids generating a sympatric distribution of South African *Menneus* and *Deinopis* (Fig. 4). These vicariant and sympatric divergences generated three distinct clades: Western Hemisphere *Deinopis,* Eastern Hemisphere *Deinopis* + *Asianopis*, and *Menneus.*

Results from the biogeographic analyses supported LDD and vicariant divergences within Eastern Hemisphere *Deinopis* + *Asianopis* (Fig. 4)*.* There are two Mexican deinopid clades that diverge from the South African *Deinopis,* one in the Upper Cretaceous and a second in the Eocene. In the absence of UCE data for the earlier diverging Mexican clade, the mechanism and direction of the dispersal was uncertain. Following the divergence of Mexican and South African *Deinopis*, there were two subsequent vicariant divergences: (1) African and Malagasy *Deinopis* diverged, (2) Malagasy *Deinopis* diverged from India + Asian *Asianopis*. Finally, there was a single LDD event from Asia to Australia resulting in the Australian *Deinopis* clade (Fig. 4). Both the UCE only (*A. liukuensis* absent) and UCE + COI concatenated biogeographic analyses indicated an ancestral range of *Asianopis* + Australian *Deinopis* in Asia. The inferred ancestral range of Australian *Menneus* was South Africa + Australia and diverged from South Africa around 56 Ma (24.22–94.35, Ma HPD 95%) (Fig. 4, Table 1). Since the date of divergence post-dates when Africa split from Australia^53^, *Menneus* likely dispersed via LDD from South Africa to Australia (Fig. 4). The 95% highest posterior density (HPD)^54^ varied widely across nodes, and is likely a consequence of having only one fossil to date the phylogeny (Table 1, Fig. S5).

### Ancestral state reconstruction

**Figure 5:**
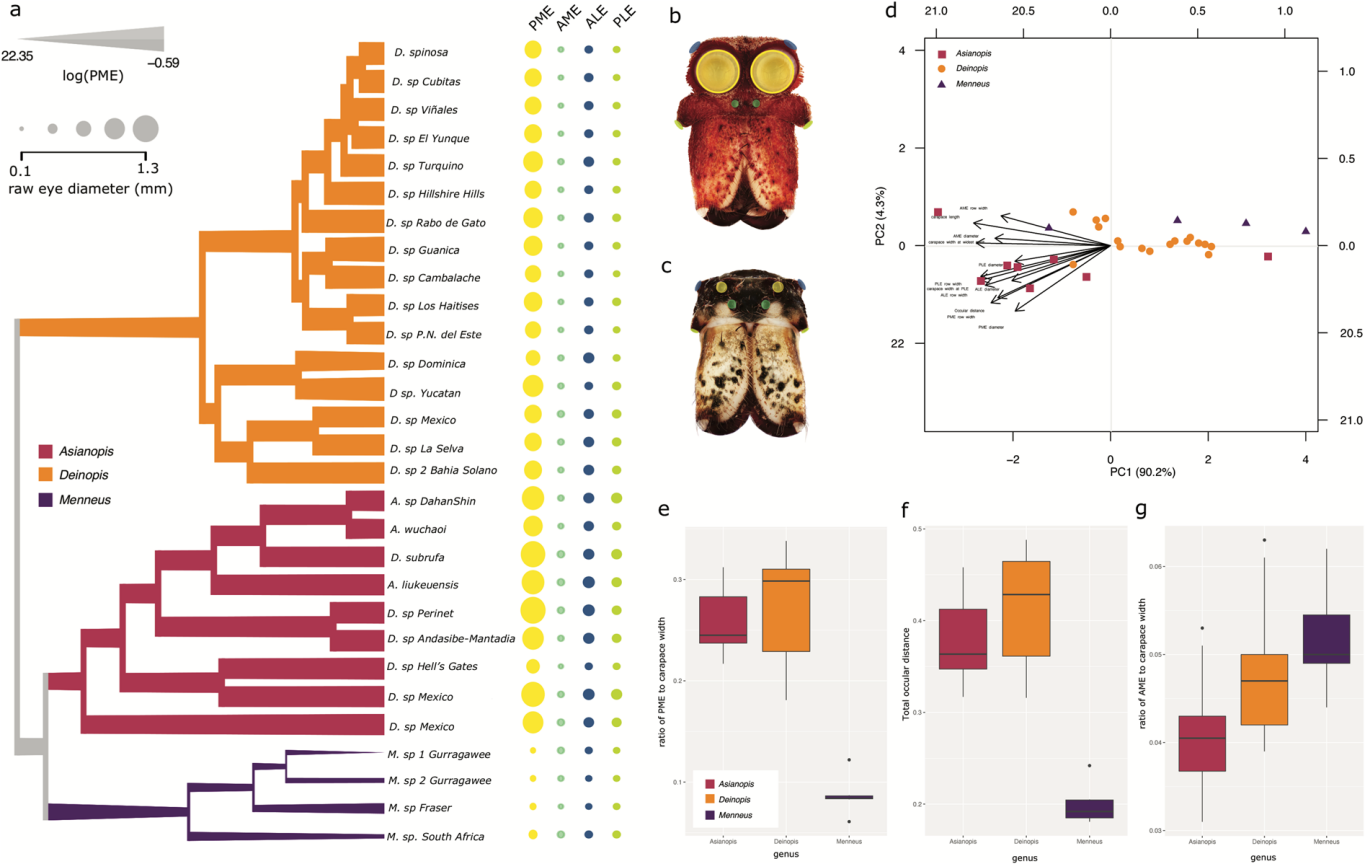
Summary of eye size data. (**a**) MCC phylogeny with branch width representing the log of PME to carapace ratio. The size of the dots represents raw eye diameter (mm) and are colored by eye type: PME (yellow), AME (blueish green), PLE (blue), ALE (yellowish green). (**b**) *A. subrufa* and (**c**) *M. sp Gurragawee* with eyes colored by type- also represented in dot phylogeny. (**d**) pPCA biplot of deinopid genus clusters (n = 29) based on carapace and eye dimensions. Clades, boxplots, and pPCA are colored by genus: *Asianopis* (magenta), *Deinopis* (orange); *Menneus* (purple). Boxplots represent (**e**) PME diameter scaled to carapace width, (**f**) total ocular distance, and (**g**) AME scaled to carapace width.

There was a single shift in PME size, from large to small, in Deinopidae at the point of *Menneus* diversification (Fig. 5a–c, Supplementary Fig. S7). PME size was highly conserved and had a strong phylogenetic signal estimated D (− 2.11). The average PME diameter (scaled to carapace width at the PLEs) and total ocular distance of adult females were significantly larger for *Deinopis* and *Asianopis* compared to *Menneus* (Fig. 5; Supplementary Fig. S8). The tests of PME/carapace ratio and raw averages for the Mann–Whitney non-parametric test of mean difference between large and typical size PMEs were both significant p < 0.0001. In the phylogenetic principal component analysis (pPCA), the first principal component (PC) (PC1, 90.2% of the variance) was strongly affected by carapace measurements: length (− 0.983), width at widest point (− 0.963), width at PLE (− 0.934). The second PC (PC2, 4.3% of the variance), with a negative loading on PME diameter (− 0.469) and PME row width (− 0.410) and positive loadings on anterior median eye (AME row width (+ 0.214) and carapace length (+ 0.163) (Supplementary Table S2). The directionality of the vectors in the biplots are close together, indicating these traits are highly positively correlated (Fig. 5d). PC1 reflected body size whereas PC2 reflected PME and AME size and eye row width; however, while the three genera generally clustered in the pPCA, the combination of morphological characters used did not strongly differentiate the three genera. There was no significant difference between PME size and total ocular distance between *Deinopis* and *Asianopis*; however, *Menneus* PMEs and total ocular distances were significantly smaller (Fig. 5e,f). Average AME size was highest in *Menneus,* although this was not significant (Fig. 5g).

## Discussion

### Monophyly of Menneus and evidence for secondary loss of enlarged PME

Phylogenetic data (UCE and UCE + COI datasets) strongly support the monophyly of *Menneus,* whereas *Deinopis* and *Asianopsis* are rendered paraphyletic (Figs. 3, 4)*.* These findings contradict the prevailing taxonomic hypothesis of the genus *Deinopis* (MacLeay 1839), as well as the more recently proposed new genus *Asianopis*^16^*.* Instead, all analyses support a deep divergence between Western Hemisphere *Deinopis* and Eastern Hemisphere deinopids (*Deinopis* + *Asianopis*), and the UCE and UCE + COI phylogenies support divergence between *Menneus* and Eastern Hemisphere *Deinopis.* Recent taxonomic studies have transferred a number of the Asian *Deinopis* species into *Asianopis*^16,55,56^; however, Australian and Malagasy *Deinopis* have yet to be moved into *Asianopis.* Based on the geographic and taxonomic coverage of the family in this study, including the deep divergences between the Eastern Hemisphere *Deinopis* + *Asianopis* clade, the *Menneus* clade, and the paraphyly of *Deinopis* with respect to *Asianopis*, we propose the transfer (see Taxonomic Section below) of the Eastern Hemisphere *Deinopis* into *Asianopis* Lin et al.^56^, thus retaining the three genera within Deinopidae: *Deinopis* in the Western Hemisphere, *Asianopis* in the Eastern Hemisphere, and *Menneus* (Simon 1876). Based on the phylogenetic hypothesis, we also propose the transfer of a number of Central American and Mexican *Deinopis* to *Asianopis.* Although these results highlight the need for further taxonomic treatment of the family and the formulation of a new diagnosis of *Asianopis*, we see no other working alternative that would adequately reconcile these phylogenetic results with the current taxonomy. That is, considering *Asianopis* and *Menneus* as junior synonyms of *Deinopsis* would fail to appropriately acknowledge the family’s phylogenetic structure, complex and worldwide biogeography, remarkable morphological divergence, and lineage divergence over major evolutionary time scales. The error bars around all major divergences are large, which is likely due to limited data and the use of a single fossil and broad priors to date the phylogeny. Still, major divergences are consistent with the previously published dated phylogeny of deinopids^15^.

We found support for a single reduction of PME size in deinopids (Fig. 5a, Supplementary Figs. S7, S9), indicating enlarged PMEs were an ancestral deinopid trait with subsequent size reduction in *Menneus.* Although it has been postulated that *Deinopis* species evolved large PMEs through adaptation to hunt exclusively at night, leaving *Menneus* to hunt at dusk and twilight, empirical testing of this hypothesis has been limited to two Australian species: *D. subrufus* and *M. unifasciatus*^57^. Extensive field observations indicate that *Deinopis* and *Menneus* hunt at the same time in low-light conditions (pers. obsv.). The observation that both genera are hunting at the same time and in the same locality, using the same hunting strategy, raises the question—what are the potential ecological shifts and evolutionary tradeoffs driving morphological divergence? The reduction in *Menneus* eyes size could be interpreted as regressive evolution. Regressive evolution, the loss or reduction of non-functional, formally adaptive characters, has been repeatedly observed in the eyes of subterranean^23,58^ and cave dwelling organisms^59^, animals of the deep ocean, and among species that make the switch from diurnal to nocturnal habitats^60–62^. Alternatively, although PME size are reduced in *Menneus*, they are not non-functional. Analyses of the anatomical structures have indicated *Deinopis* PMEs are more sensitive than *Menneus*^17^; however the extent of the visual sensitivity between the two genera remain largely unknown and understudied.

Among organisms where regressive evolution has been observed, there are three primary, not mutually exclusive, hypotheses that seek to explain character reduction: (1) relaxed selection or neutral mutations—the relaxation of selection leading to reduction (e.g. loss of eyes, reduction of eye size)^63–65^; (2) natural selection, in which reduced visual structures or the absence of eyes are advantageous^66–68^; (3) indirect selection, pleiotropy, or ‘genetic hitchhiking’, in which the gene related to trait reduction is linked to other adaptive trait(s) (e.g. olfaction or taste)^29,69–71^. *D. spinosa* has been shown to use auditory cues to capture their prey^7^. Future studies could, for example, explore whether *Menneus* has evolved morphological and/or behavioral characters that are advantageous for capturing aerial prey (e.g. additional sensitive vibratory sensors such as trichobothria). Furthermore, in *D. spinosa,* individuals use their large eyes to hunt cursorial prey, which ultimately increases their dietary breadth^14^; however, enlarged PMEs are metabolically costly. Stafstrom et al.^72^ found that there was a potential tradeoff between brain size and PME size. *Deinopis* PMEs are more complex^13,73^ and twice as sensitive compared to *Menneus* PMEs^17^. We postulate that relaxed selection in environments where such eyes do not offer major fitness benefits may explain this hypothesized secondary loss. Explicit tests of differential time and mode of hunting and evaluation of the underlying genetics of phenotypic differences may help tease apart potential evolutionary mechanisms. Finally, we propose an alternative hypothesis in which *Menneus* have far more sensitive PMEs than spiders with similarly sized eyes. If there was strong selection for increased eye size, neural connections, and light detection mechanisms in *Deinopis*, an energy cost could be spared by reducing eye size but retaining the high sensitivity. In that case there is a secondary reduction in eye size, but not, a reversal to the ancestral spider condition. Future detailed studies on receptor function, light sensitive cells, and visual/neural system communication could find that taking that advanced eye and making it smaller in *Menneus* would be advantageous even with certain anatomical simplifications from *Deinopis*. Deinopids have tremendous potential as a unique system for testing these alternative hypotheses and for studying visual evolution.

### Early Gondwanan vicariance followed by long-distance dispersals

The disjunct geographic distributions of deinopids can be attributed to Upper Cretaceous vicariance during the breakup of West Gondwana, followed by subsequent transoceanic dispersal events. Initially formed around 500 Ma, Gondwana, the southern portion of the supercontinent Pangaea, began breaking away in the Middle Jurassic around 170 Ma^74–77^. However, the continents did not all break apart in succession. Instead, continents were simultaneously separating, with phases of these overlapping divergent events lasting tens of millions of years. After the initial separation, land bridges persisted between continental masses, providing passageways for biotic exchange^78^. Even amongst taxa with low vagility, there is mounting evidence for early Gondwanan vicariance followed by subsequent LDD events in taxa, including plants^79–82^, cave shrimp^83^, freshwater fish^84^ and arachnids^85,86^. Of course, in the epochs immediately following continental breakup, the distances between major landmasses were shorter and thus dispersal among these would be more likely than among the continents in their current positions. Following these early divergences driven by the breakup of West Gondwana, we concluded that subsequent deinopid diversification occurred via long-distance, trans-oceanic dispersal events.

### Dispersal into Asia and Australia

With a largely tropical and sub-tropical range, deinopids do not follow the typical anti-equatorial distributions across the Southern Hemisphere found in many Gondwanan lineages^87–93^; nonetheless, vestiges of the ancient Gondwanan connections are still reflected in the biogeographic history of deinopids. Following the divergence from *Menneus,* Eastern Hemisphere *Deinopis/Asianopis* dispersed from South Africa to Madagascar where they subsequently spread into Asia. The *Deinopis/Asianopis* lineage dispersed and diversified throughout Asia, with later dispersal to Australia via Indonesia. The timing of the divergences and biogeographic analyses suggest that *Deinopis* may have arrived in Asia from India after India collided with Southeast Asia in the Eocene (Out of India hypothesis). The duration of the hypothesized IndoMadagascar landmass is debated. These may have separated as early as 130–125 Ma, but remained connected to India until around 84 Ma^94,95^ after which it is thought that fracture zones, plateaus, periodically emerged and facilitated stepping stone dispersal between Madagascar and India^96^. Such hypothesized stepping stone dispersal from Africa into Asia via India and Madagascar has been found in taxa with low vagility^97–99^. Alternatively, *Deinopis* could have dispersed through Northern Africa and Eurasia followed by subsequent extinction similar to sand scorpions^100^; however, our molecular phylogeny shows that the Madagascar clade is more closely related to Asia and India than Africa, consistent with the IndoMadagascar plus stepping stone hypothesis. Because this molecular dataset only included COI data for *A. luikenisis* from China and Taiwan, further sampling throughout India and Southeast Asia will be required to more rigorously test this dispersal hypothesis. Alternatively, these data support the hypothesis that *Menneus* dispersed to Australia via a single, trans-oceanic dispersal event, although divergence times of Australian and African *Menneus* post-date an Out of India hypothesis. With 37% of the total 46 described deinopid species in the Eastern Hemisphere (putative and genetic transfers), much of Africa, Asia, and Indomalaya remains under-sampled. More extensive sampling is required, particularly of Madagascar and Indomalaya, to more completely understand *Menneus* biogeographic history.

### Crossing Wallace’s line

Wallace’s line is one of the most striking separations of biomes in the world. Wallace^101^ posited that the fauna of South America and Africa, despite being separated by the Atlantic Ocean, were more similar than the faunas of Asia and Australia. Based on our results, deinopids dispersed from Africa to Asia and then from Indomalaya to Australia 91–52 Ma (Fig. 4), which may reveal intriguing implications of dispersal across Wallace’s line. Furthermore, the Australian *Deinopis* diverged from its sister Indomalayan clade around 72 Ma (Fig. 4). This divergence pre-dates the Indo-Australian archipelago (IAA) and a deep sea and a large trench separated the two regions until around 40 Ma when islands began forming^102^. Recently the IAA has been discovered to be a more common route of dispersal than previously predicted in terrestrial animals^102–104^. We postulate that upon broader sampling in this region, we may find range expansions across the IAA in the Late Eocene to Early Miocene when landmasses were forming^102^.

### South Africa, Madagascar, and the Neotropics

We found evidence for at least two trans-oceanic dispersals of South African *Deinopis*. First, Malagasy *Deinopis* diverged from South African *Deinopis* in the Paleocene, post-dating Lower Cretaceous continental drift and vicariance. Madagascar separated from Africa and moved southeastward in the Middle to Early Late Jurassic (160–155 Ma)^105,106^ and was one of the first of the Gondwanan landmasses to break away from the supercontinent. Madagascar was in its final position relative to Africa approximately 120 Ma (118–130 Ma)^107,108^. The relative contributions of Gondwanan vicariance and long-distance dispersal among Malagasy lineages remain a source of debate^96,109–111^. Madagascar’s biodiversity primarily comprises endemic flora and fauna that have either been present since Gondwana or were a product of long-distance dispersal events in the Cenozoic, mostly from Africa (400 km away), but including all major biogeographic regions^96^.

Second, we found evidence for at least one trans-oceanic long-distance dispersal between South Africa and Mexico in the Eocene. Discoveries of such extreme trans-oceanic dispersals are becoming increasingly common^41,99,112^ and can be attributed to salt-water tolerance^113^, long-distance flight^114–116^, and wind-dispersal in plants^99^ and ballooning spiders^117,118^. Exchanges between Africa, the Western Indian Ocean, and the Neotropics have been especially well documented in tropical flora^119–125^. Dispersal events from the Neotropics to Africa are facilitated by westerly winds^125,126^, whereas dispersals from Africa to the Neotropics, the rarer of the two dispersal scenarios^125^, typically follow the trade winds^126^. While there was at least one dispersal event between Africa to the Mexico, further sampling of Eastern Hemisphere deinopids in the Neotropics is necessary to rigorously test dispersal hypotheses.

## Conclusions

Deinopids have a rich biogeographic history characterized by complex and ancient patterns of vicariance coupled with long distance dispersal since their emergence in the Cretaceous. While LDD has played an important role in shaping the distributions of deinopids, these events are not so prevalent as to completely obfuscate ancient Gondwanan signatures. The evolutionary history of Deinopidae provides fascinating examples of eye size reduction and unexpected trans-oceanic dispersals. Whereas most studies in eye size evolution are troglomorphic adaptations in the form of reductions/loss of eyes, here, we document an example of a single reversal to ‘normal’ sized eyes from the uniquely derived condition of grossly enlarged PMEs in ogre-faced spiders. The PME size reductions in *Menneus* provides a framework for considering and testing hypotheses of the potential selective forces that might be driving these divergent morphologies.

### Taxonomic section

Based on phylogenetic structure and biogeographical distributions we propose the following new combinations (* indicates those taxa included in this study): *Asianopis anchietae* (Brito Capello^127^) NEW COMBINATION; *Asianopis aspectans* Pocock^128^ NEW COMBINATION; *Asianopis aurita* Pickard-Cambridge^129^ NEW COMBINATION; *Asianopis camela* Thorell^130^ NEW COMBINATION*; Asianopis cornigera* Gerstaecker^131^ NEW COMBINATION; Asianopis cylindrica* Pocock^132^ NEW COMBINATION; *Asianopis fasciata* Koch^133^ NEW COMBINATION; *Asianopis fasciculigera* Simon^134^ NEW COMBINATION; *Asianopis giltayi* Lessert^135^ NEW COMBINATION; *Asianopis guineensis* Berland & Millot^136^ NEW COMBINATION; *Asianopis kollari* Doleschall^137^ NEW COMBINATION; *Asianopis labangan* Barrion-Dupo & Barrion^138^ NEW COMBINATION; *Asianopis longipalpula* Strand^139^ NEW COMBINATION; *Asianopis luzonensis* Barrion-Dupo & Barrion^138^ NEW COMBINATION; *Asianopis madagascariensis* Lenz^140^ NEW COMBINATION; *Asianopis mediocris* Kulczyński^141^ NEW COMBINATION; *Asianopis ornata* Pocock^142^ NEW COMBINATION; *Asianopis ravida* Koch^143^ NEW COMBINATION; *Asianopis reticulata* Rainbow^144^ NEW COMBINATION; *Asianopis schomburgki* Karsch^145^ NEW COMBINATION; *Asianopis schoutedeni* Giltay^146^ NEW COMBINATION; Asianopis subrufa* Koch^143^ NEW COMBINATION; *Asianopis tabidus* Koch^133^ NEW COMBINATION; Asianopis unicolor* Koch^143^ NEW COMBINATION.

## Methods

### Taxon sampling

We collected a total of 42 deinopid individuals (29 *Deinopis* and 13 *Menneus*) from the east coast of Australia in January 2019 using standard aerial search and vegetation beating methods described in Coddington et al.^147^. Most of the specimens were collected at night as deinopids are highly cryptic and are most easily spotted when their nets reflect a blue hue under headlamp lights. The specimens were fixed in 95% ethanol in the field and kept at − 20 to − 80 °C in the lab. Collaborators also provided additional *Deinopis* specimens from Western Australia, Taiwan, Mexico, Madagascar, South Africa, and Colombia.

### DNA extractions, sequence generation, and data processing

Detailed methods for molecular methods, sequence editing, and read processing are available in the Supplemental Methods S1. We extracted DNA from leg tissue for 65 individuals using the OMEGA BIO-TEK E.Z.N.A. DNA extraction kit. We amplified COI: cytochrome c oxidase subunit I, a mitochondrial locus. We obtained COI sequence data for seven species from GenBank for outgroups, following^148^. We included existing deinopid COI data available on DRYAD^149^ and Genbank from Lin et al.^16^ for additional global *Deinopis* samples and *Asianopis* samples, respectively. See Supplementary Table S3 for full taxon sample lists, GPS locality information, and Genbank accession numbers. Sequences were aligned in MAFFT^150^ and then edited by eye and checked for stop codons in Mesquite^151^.

For UCE data, we sampled at least two individuals from each major clade and their geographic representation, including from African *Menneus*, Australian *Menneus*, African *Deinopis*, Australian *Deinopis*, Malagasy *Deinopis*, *Asianopis*, North and Central American *Deinopis*, and Caribbean *Deinopis*. UCE libraries were hybridized to the Spider probeset^148^ 150 bp paired-end reads were sequenced on the HiSeq4K at the University of California Davis DNA Technologies (See Supplemental Methods S1 for more detail). We obtained UCE data for four outgroup species from Kulkarni et al.^148^ (NCBI Sequence Read Archive, BioProject PRJNA575576). UCE and COI matrices were concatenated in AMAS^152^ to generate the UCE + COI combined dataset.

### Phylogenetics

We inferred phylogenetic trees for the four molecular datasets: COI-only (258 deinopid individuals, four outgroup individuals; 1279 base pairs), UCE-only (40 deinopids, four outgroup individuals; 75% occupancy matrix; 1018 loci), COI + UCE concatenated, and COI with a UCE backbone phylogeny using Maximum Likelihood^153^ and coalescence-based methods^154^. In IQ-TREE 2^153^, we implemented the built-in model finder to consider invariant site and Gamma rate heterogeneity under the *greedy* strategy with the AICc criterion to determine molecular substitution models for each codon position. The COI dataset was partitioned by codon using the GTR + I + G and the UCE dataset was partitioned by locus using the GTR model. We ran four separate analyses on each dataset with 1000 pseudoreplicates of ultrafast bootstrapping^155,156^ on the codon-partitioned COI and COI-backbone datasets, and the loci-partitioned UCE, COI + UCE concatenated datasets. We generated trees from individual UCE loci using IQ-TREE. These trees were used to infer a coalescence-based species tree in IQ-TREE and ASTRAL-II v5.7.1^154^.

### Tree topology tests

We implemented tree topology tests in IQ-TREE 2^153^ to evaluate the relationships of *Asianopis, Deinopis,* and *Menneus,* and to further assess the monophyly of these three groups. We tested the unconstrained tree and four alternative tree topologies (Supplementary Fig. S1) in IQ-TREE using the RELL approximation^157^, which uses bootstrap proportions, p-values with the Shimodaira-Hasegawa test^158^ and AU test^159^, and expected likelihood weights^160^. Using the UCE dataset, we inferred species trees in IQ-TREE for the datasets constrained to four alternative hypotheses (1) *Menneus* is paraphyletic (clades = New World *Deinopis*, Australian *Deinopis* + *Menneus*; African *Deinopis* + *Menneus*; Malagasy *Deinopis*; *Asianopis*); (2) *Menneus* is monophyletic and *Deinopis* and *Asianopis* form a clade (clades = *Menneus*, all *Deinopis* + *Asianopis*); (3) *Menneus*, New World *Deinopis*, Old World *Deinopis*, and *Asianopis* are each monophyletic; (4) *Menneus*, all *Deinopis*, and *Asianopis* are monophyletic (clades = *Menneus*, *Deinopis*, *Asianopis*) (Supplementary Fig. S1).

### Molecular dating

We implemented MCMCTree in the PAML v4.8^161^ package on the UCE only dataset to estimate divergence times within Deinopidae. Prior to the analyses, we removed redundant subspecies so that only one individual per species (with the least amount of missing data) remained. To set up the model and model parameters, we assigned a birth–death model for the tree and an UCLN clock model (uncorrelated relaxed clock). A GTR substitution model was used with flat Dirichlet prior distribution. Previous phylogenetic dating analyses have used the fossil *Palaeomicromenneus lebanensis* to calibrate the stem of Deinopidae^15,148,163^. However, the taxonomy of this fossil is dubious^164^, and it has been transferred into the extinct family Salticoididae^165^; therefore we did not use this fossil as a calibration point in the dating inferences. Instead, we employed a molecular calibration point from recent phylogenetic reconstruction on Araneomorphs^166^ to conservatively use maximum age of 150 Ma as a soft constraint for the Deinopidae crown. We used a single fossil, *Seppo koponeni* to calibrate a soft constraint with the minimum root age of Deinopidae + outgroups of 132.9 and a maximum of 250 Ma. We ran two independent Markov chain Monte Carlo (MCMC) runs for 20,000 samples, resampling every 10,000 iterations for a total of 200,000,000 iterations with a burn-in set to discard the first 20,000,000 iterations. Outputs were assessed for stationarity in Tracer v1.7.1^167^ and visualized using FigTree v1.4.4^168^.

### Ancestral range estimates

We estimated the ancestral ranges of deinopids and performed biogeographic analyses in BioGeoBEARS^169^ using the UCE-only MCC tree inferred in MCMCTree. We used eight isolated biogeographic areas including Africa (F), Asia (A), Australia (U), Neotropics (S), Nearctic (N), Madagascar (M), India (I), and the Caribbean (C), five of which were based on updated zoological regions of Holt et al.^170^ and on the most recent global biogeographic studies on spiders^171^. These regions were also selected due to their high endemism, geologic histories, and land availability. We included a time slice model based on the breakup of the continents following descriptions in Seton et al.’s^172^ of the historical geology and modeled after recent invertebrate dispersal models^43,44^ (Supplementary Table S4; See Supplemental Methods S1). We tested these models under the Dispersal‐Extinction‐Cladogenesis (DEC) and with the (+ *j*) parameter, which considers for jump dispersal or founder event speciation^169,174^. We compared Akaike information criterion (AIC) and relative likelihood scores across all models (a natural log of 2 was considered significant)^175^. We used the MCMCTree-generated UCE and UCE + *COI* dated phylogeny with outgroups pruned to only include Deinopidae specimens.

### Ancestral state reconstruction and eye measurements

Measurements of adult female specimens were taken with a Leica M205 C scope using the micrometer scale tools in the Leica Application Suite (LAS Version 4.13.0) (Supplementary Table S5; See Supplemental Methods S1). We measured the total ocular distance and individual diameters of PME, anterior median eye (AME), anterior lateral eye (ALE), posterior lateral eye (PLE) diameter for all adult females. We also measured carapace length, width, and carapace width at the PLEs. A Mann–Whitney non-parametric test of mean difference was used to determine whether there was a statistically significant difference between large and small PME. We used a phylogenetic principal components analysis (pPCA) in R using the function *phyl.pca* in the R package phytools^176^ to determine if eye and carapace size, ten and three characters respectively, were partitioned among the three genera: *Deinopis, Asianopis,* and *Menneus.* We used a pruned MCC tree, which included 29 species throughout the tree that were absent of missing data. Data points included carapace length and width, eye diameter of all four types of eyes (PME, AME, PLE, and ALE) and the eye row width across each of these. We also calculated total ocular distance, the sum diameter of all eight eyes. We generated biplots in R of the scores of the 23 total traits obtained from the pPCA.

We tested the phylogenetic signal of the binary trait for eye size, “large” for *Deinopis* and “small/typical” for *Menneus,* and for the outgroups*.* We calculated D, which is a permutation-based model that measures the phylogenetic signal strength in a binary trait assuming the underlying continuous trait is evolving under Brownian Motion^177^ and was appropriate for eye size, which is a variable and potentially continuous trait within each genus.

We mapped the states for discrete characters, large and small PME, along the species level MCC phylogeny using the *ace* function in *ape*^178^ and in *phytools*^176^. To estimate ancestral states for discrete PME characters, we employed a continuous time Markov chain (Mk) model to give character probability distributions of ancestral states at internal nodes in the tree. We also used stochastic character mapping, an MCMC approach to sample character histories from their probability distributions.

## Supplementary Information

Supplementary Information.

## Data Availability

The datasets generated during and/or analyzed during the current study are available from the corresponding author on reasonable request. The UCE dataset generated and analyzed during the current study have been deposited at NCBI Short-Read Archive (https://www.ncbi.nlm.nih.gov/sra) as BioProject PRJNA802018. All COI sequences obtained in this study have been deposited at NCBI GenBank (https://www.ncbi.nlm.nih.gov/genbank/) with the accession numbers OM480748-OM480975.
