# Supplementary material for: Biogeography and eye size evolution of the ogre-faced spiders
Source: Sci Rep. 2022 Oct 22;12:17769. doi: 10.1038/s41598-022-22157-5 (PMC9588044; doi:10.1038/s41598-022-22157-5)
Supplement: Supplementary file 1 — Supplementary Information. [file 41598_2022_22157_MOESM1_ESM.zip › Supplemental_Material/Supplementary_Figures.docx]

**Supplementary Material**

**Biogeography and eye size evolution of the ogre-faced spiders**

**Lisa Chamberland^1*^, Ingi Agnarsson^2^, Iris L. Quayle^1^, Tess Ruddy^3^, James Starrett^1^, and Jason E. Bond^1^**

^1^Department of Entomology and Nematology, University of California Davis, Davis, CA 95616, USA

^2^Faculty of Life and Environmental Sciences, University of Iceland, Sturlugata 7, 102 Reykjavik, Iceland

^3^Vassar College, Poughkeepsie, NY 12604

*Corresponding author

Dr. Lisa Chamberland

lchamberland@ucdavis.edu

**Supplementary Figure S1.** Tree topology hypothesis implemented in IQtree. (H0) Null hypothesis, unconstrained phylogeny with New World *Deinopis*, *Menneus*, three Old World *Deinopis* clades and *Asianopis*. (H1) *Menneus* and *Deinopis*+*Asianopis* monophyletic. (H2) Australian deinopids and South African deinopids monophyletic. (H3) New World *Deinopis*, Old World *Deinopis, Menneus,* and *Asianopis* all monophyletic (H4) *Menneus, Deinopis, Asianopis* all monophyletic. Map was created using the base map form Wikimedia Commons (<https://commons.wikimedia.org/wiki/File:BlankMap-World.svg>) and country colors were modified using Adobe Illustrator (https://www.adobe.com/) (Map: Lisa Chamberland).


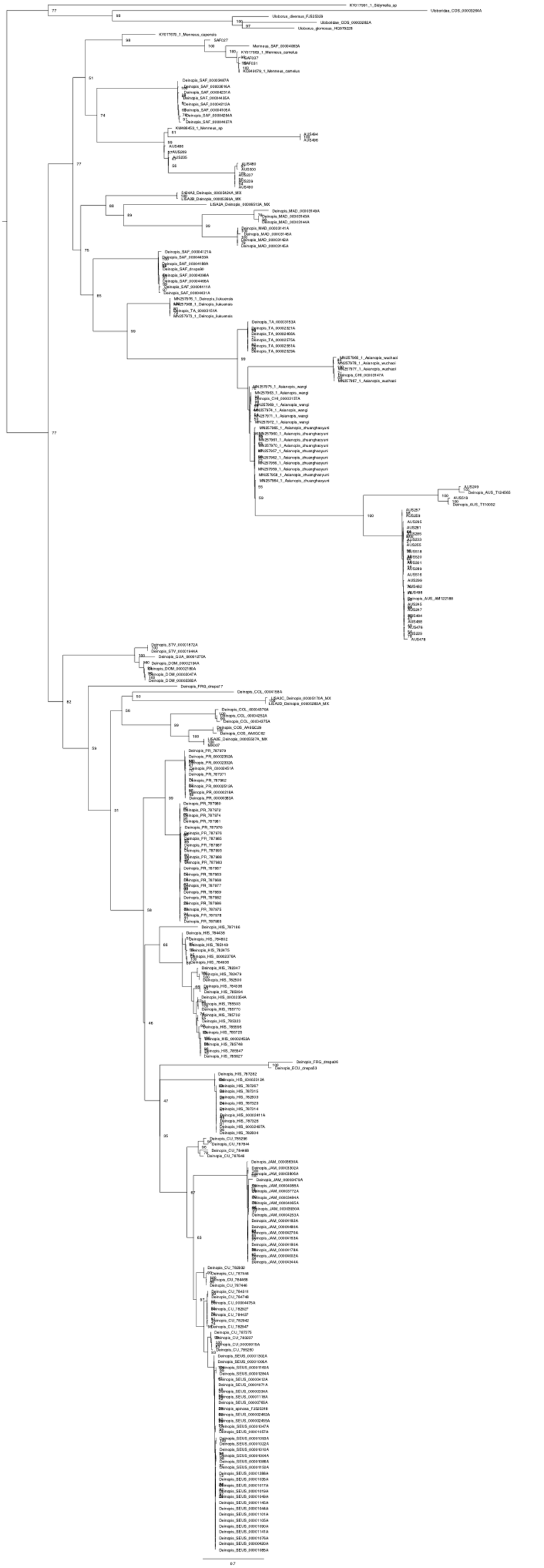


**Supplementary Figure S2**. ML phylogeny inferred in IQ-Tree of COI-only dataset. Node values indicate bootstrap support.

**
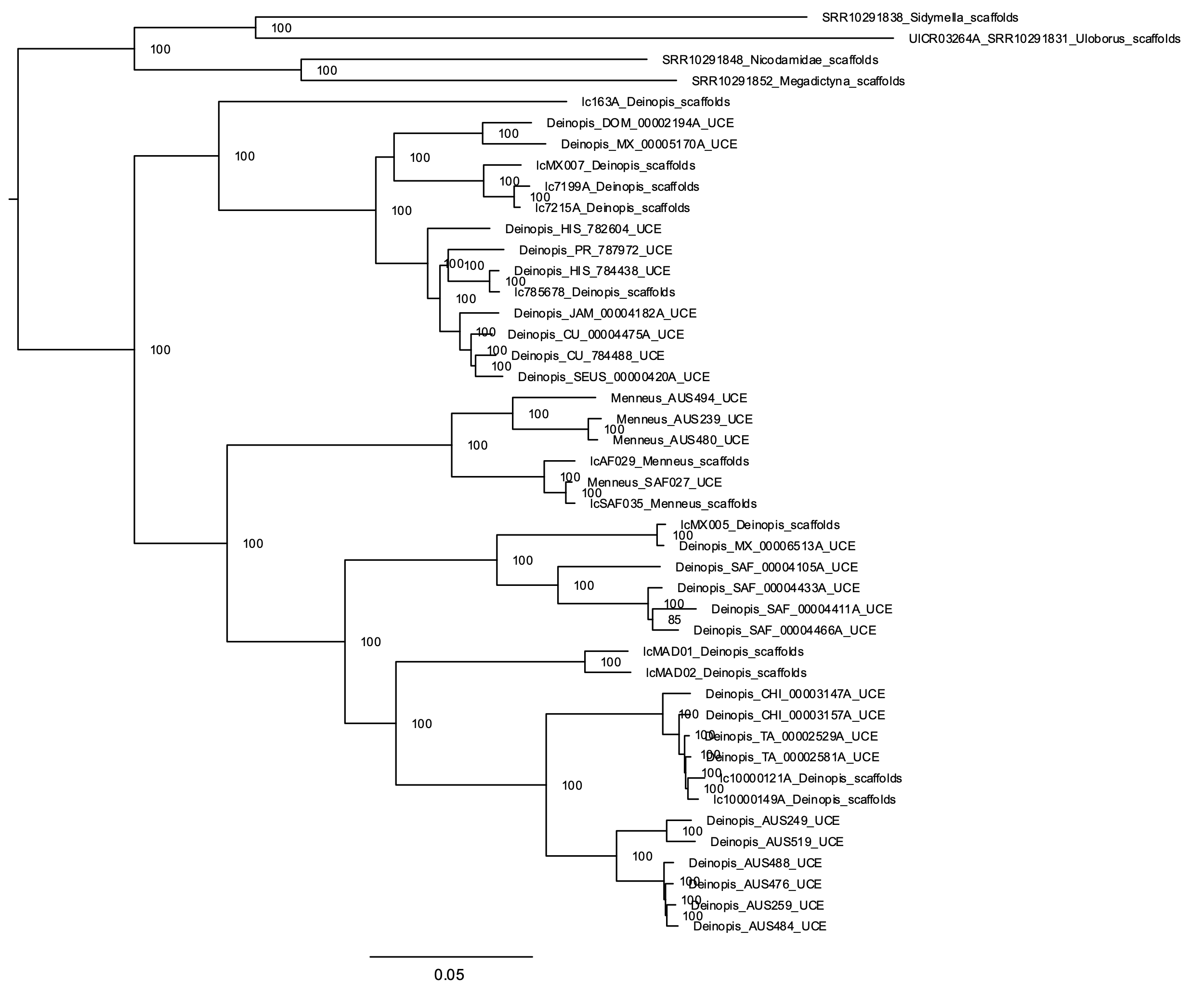
**

**Supplementary Figure S3**. ML phylogeny inferred in IQ-Tree of UCE-only dataset. Node values indicate bootstrap support.


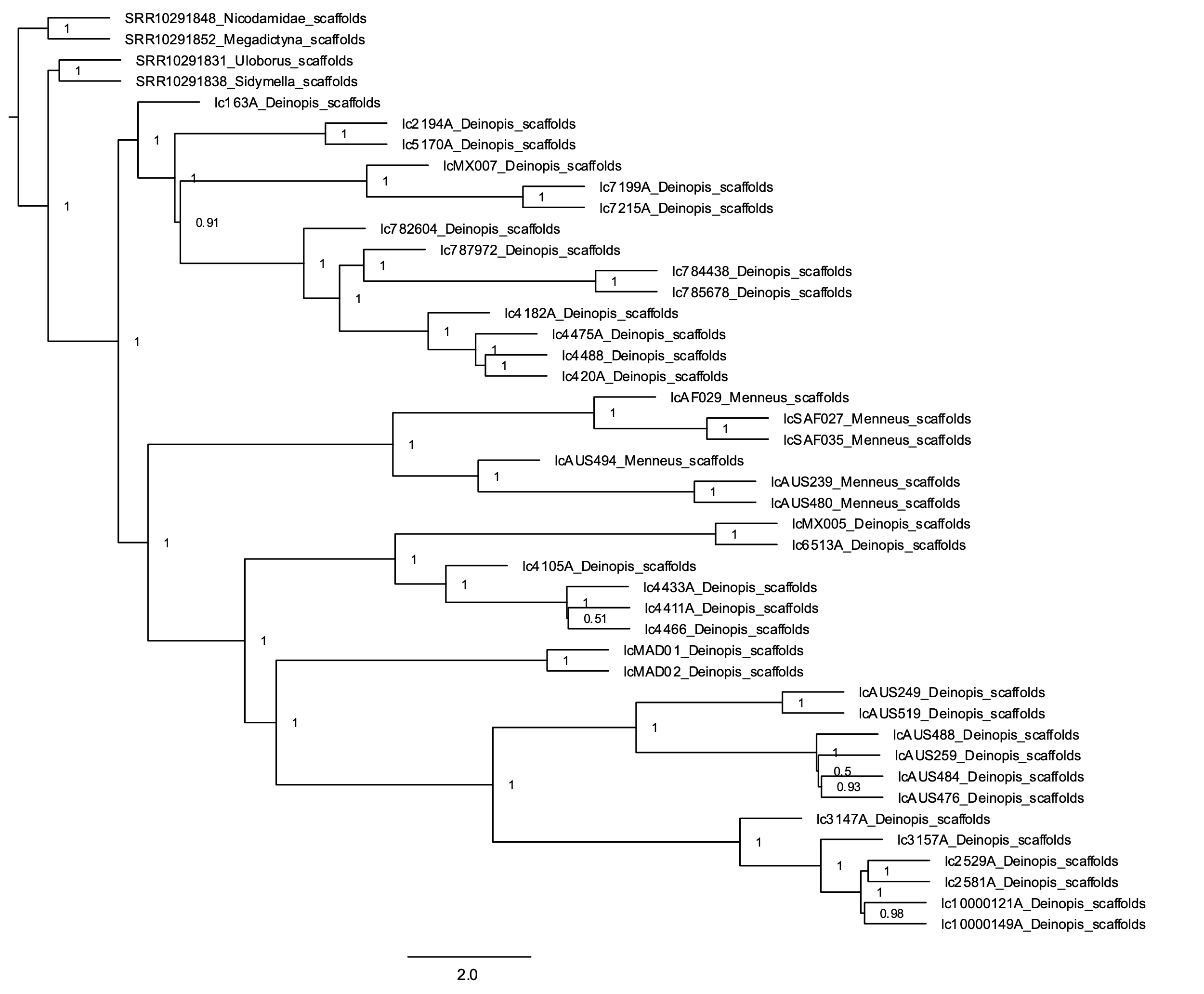


**Supplementary Figure S4.** Coalescence-based phylogeny inferred in ASTRAL of UCE-only dataset. Node values indicate posterior probability.


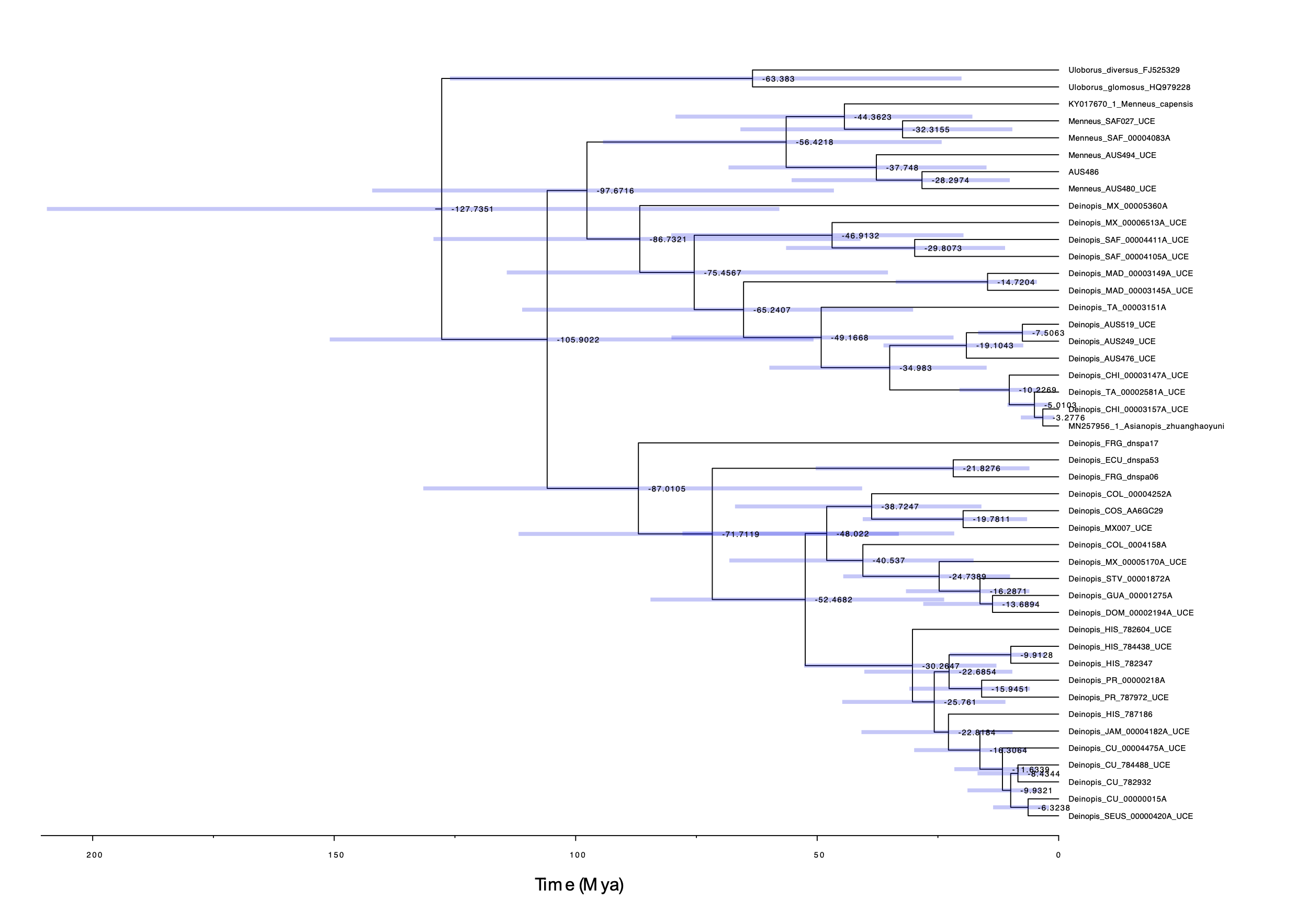


**Supplementary Figure S5.** mcmcTREE dated phylogeny of UCE+COI concatenated dataset. Node values indicate divergence times; purple bars indicate 95% HPD.

**
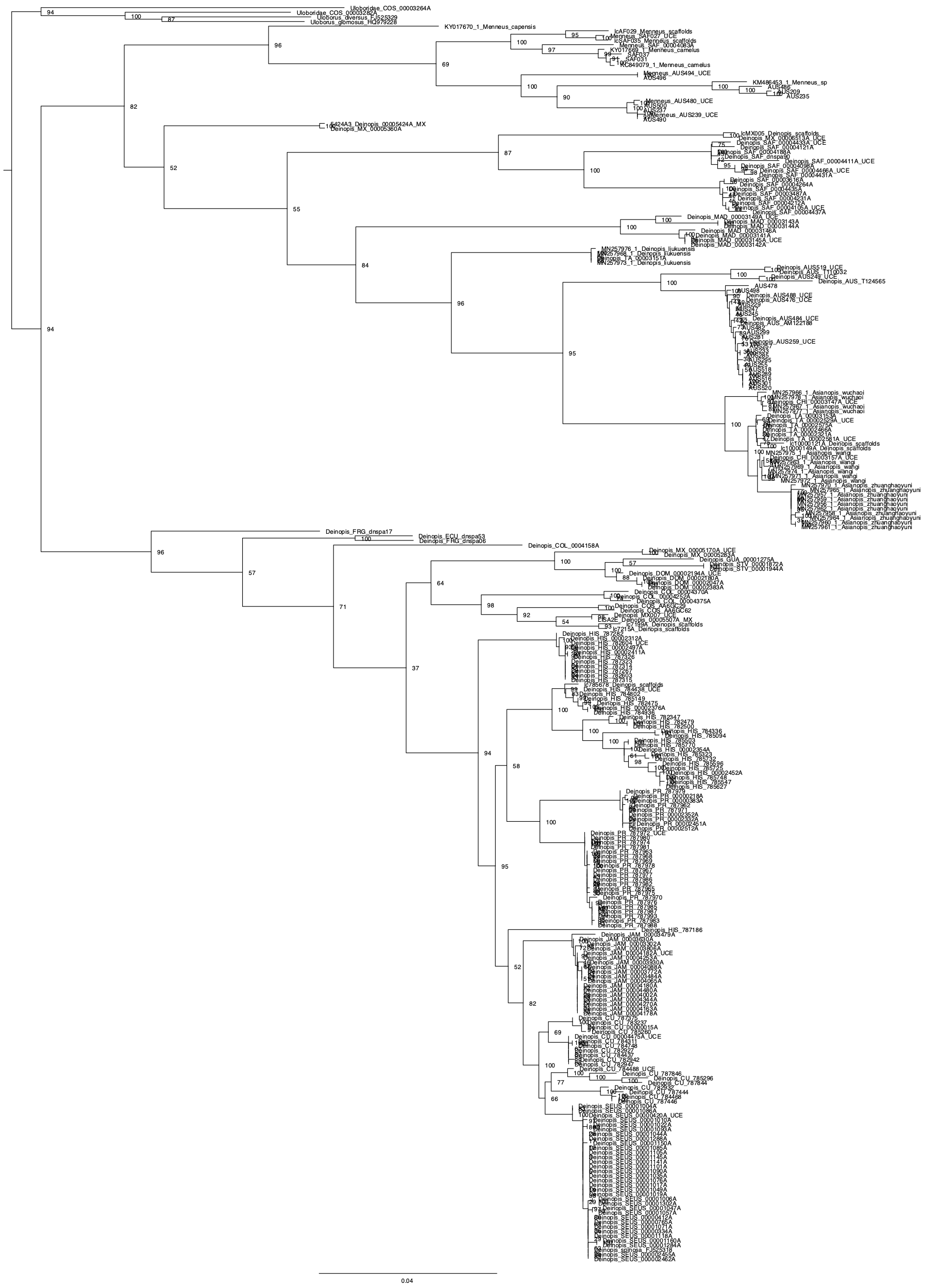
**

**Supplementary Figure S6.** ML phylogeny inferred in IQTree of UCE+COI concatenated dataset with labeled tips. Node values indicate bootstrap support.

**Supplementary Figure S7.** Ancestral character reconstruction of PME size in Deinopidae. PME here is recorded as a discrete trait: large (blue) and small (gold) PMEs.

**Supplementary Figure S8** Boxplots of PME and AME diameter and total ocular distance. Raw measurements (mm) were included and were also scaled to carapace width and carapace length.


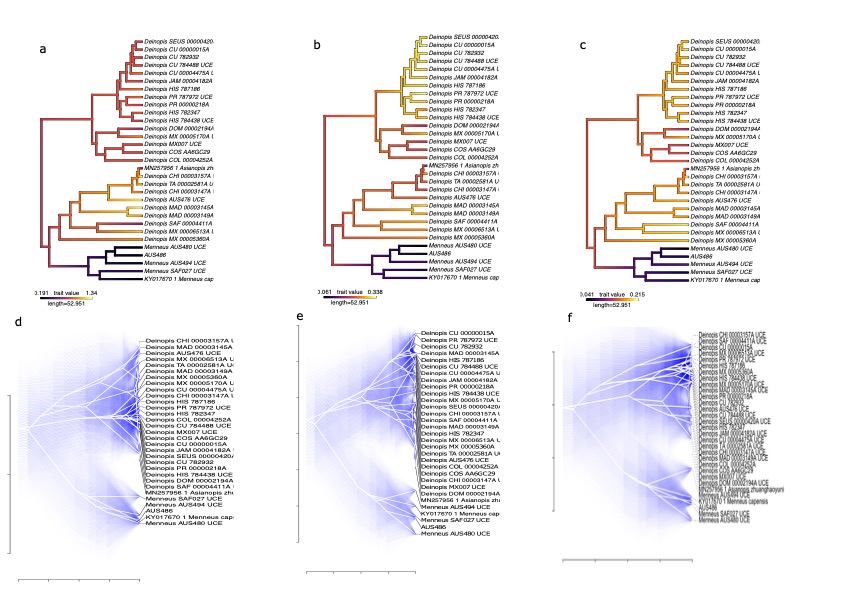


**Supplementary Figure S9.** Continuous character mapping of (a) raw PME diameter (mm), and PME diameter (b) scaled to carapace length and (c) scaled to carapace width at the PLEs, and (c) raw PME diameter. Traitgram of the phylogeny of Deinopidae projected onto the phenotypic space of PME size (d) scaled to carapace length, (e) scaled to carapace width at the PLEs, and (f) raw PME diameters (mm).
