# Supplementary material for: Biogeography and eye size evolution of the ogre-faced spiders
Source: Sci Rep. 2022 Oct 22;12:17769. doi: 10.1038/s41598-022-22157-5 (PMC9588044; doi:10.1038/s41598-022-22157-5)
Supplement: Supplementary file 1 — Supplementary Information. [file 41598_2022_22157_MOESM1_ESM.zip › Supplemental_Material/Supplementary_Methods.docx]

**Supplementary Material**

**Biogeography and eye size evolution of the ogre-faced spiders**

**Lisa Chamberland^1*^, Ingi Agnarsson^2^, Iris L. Quayle^1^, Tess Ruddy^3^, James Starrett^1^, and Jason E. Bond^1^**

^1^Department of Entomology and Nematology, University of California Davis, Davis, CA 95616, USA

^2^Faculty of Life and Environmental Sciences, University of Iceland, Sturlugata 7, 102 Reykjavik, Iceland

^3^Vassar College, Poughkeepsie, NY 12604

*Corresponding author

Dr. Lisa Chamberland

lchamberland@ucdavis.edu

#### Eye and carapace measurements

We captured eye and carapace measurements for 59 adult female deinopid individuals with a Leica M205 C scope using the micrometer scale tools in the Leica Application Suite (LAS Version 4.13.0). All measurements are in millimeters and were collected at the highest possible magnification. With the specimens positioned dorsally, we measured for carapace length and width, both at the widest point and at a cross-section through the PLE row. We measured the carapace length using the anterior edge of the carapace. In large-eyed specimens, carapace length was taken following the line of the carapace edge but measuring in the space between eyes in order to ensure a straight line. The PLE row width was measured as well as the diameter of the left PLE. To capture the anterior (face) view, we used modified 1.5 ml and Qubit vials that were cut into a cylinder to position the specimen vertically. This method ensured the specimen was stable, upright, and did not shift during measurements and photos. We measured the widths of the PME, AME, and ALE of the vertically positioned specimens. Finally, we adjusted each specimen within the cylinder to procure accurate diameters of the left PME and AME.

#### Sequence generation and data processing

1. Standard sanger sequencing

We amplified *COI*: cytochrome c oxidase subunit I using 0.4 uM of each universal DNA primers LCO1490 (5′-GGTCAACAAATCATAAAGATATTGG-3′) and HCO2198 (5′-TAAACTTCAGGGTGACCAAAAAATCA-3′). We assembled and edited the sequence data within the Chromaseq module^1^ in Mesquite v3.70^2^ with Phred and Phrap^3,4^ using default parameters.

2. Ultraconserved Elements

Following a preliminary phylogenetic analysis of the *COI* dataset, there were a number of important clades with weak nodal support, including the placement of *Menneus*. To resolve the dubious phylogenetic relationships, we generated UCEs for 40 individuals from across all major clades. We processed at least two individuals from each major clade, including from African *Menneus*, Australia *Menneus*, African *Deinopis*, Australian *Deinopis*, Malagasy *Deinopis*, *Asianopis*, South Africa, North + Central America *Deinopis*, and the Caribbean *Deinopis*.

For specimens with <250 ng of DNA, we re-extracted genomic DNA from one leg using the Blood and Tissue DNeasy kit (Qiagen) and quantified using a Qubit. Two-hundred and fifty nanograms of DNA was sheared to 300-1000 bp at the University of California Davis DNA Technologies Core (CORE facilities) with a Covaris M220 Focused-ultrasonicator. We followed the Target Enrichment of Illumina Libraries protocol (ver. 1.10, see <http://ultraconserved.org/#protocols)> for generating libraries with the KAPA Hyperprep kit (Roche) and target enrichment of libraries with MYbaits (ver. 1, Arbor Biosciences)^5^. The libraries were hybridized at 60$^{\circ}$C for 24 hours to the Spider probeset^6^ with the version 4 chemistry protocol (Arbor Biosciences). Hybridization-enriched library pools were sequenced with 150 bp paired-end reads on the HiSeq4K at the CORE facilities.

Read processing and analyses were conducted on the University of California Farm Bioinformatics Cluster. Reads were filtered and trimmed using Illumiprocessor^7^ and Trimmomatic^8^ in Phyluce v1.7.1^9^. We used the isolate option in Spades^10^ to generate assemblies de novo with the cleaned paired-end and single-end reads. Scaffolds were matched to the probe list with 65% identity and 65% coverage. We used the probe list from Maddison et al.^11^, which combines the Arachnid probeset^5,12^ and the Spider probeset^6^. MAFFT v7^13^ was used to align locus datasets, and alignments with locus occupancy minimums of 50% and 75% were generated. Finally, trimAl^14^ was implemented to perform alignment using the -automated option.

3. Combined datasets

The COI matrix was concatenated and appended to the post-trimmed 1018 loci UCE dataset. This combined dataset was used from subsequent phylogenetic analyses.

Biogeography

We estimated the ancestral ranges of deinopids and performed biogeographic analyses in BioGeoBEARS^15^ using the UCE-only MCMCTree^16^ dated phylogeny. The outgroups were removed for all biogeographic analyses, and was Deinopidae were included. We used seven isolated biogeographic areas, five of which were based on updated zoological regions of Holt et al.^17^ and based on the most recent global biogeographic studies on spiders^18^. Africa (A), Indomalaya (E), Neotropics (S), Nearctic (N), and two Madagascar (M) and Caribbean (C) additional regions because of their high endemism, geologic histories, and land availability. The Caribbean islands were also coded as a geographic region because of the disproportionate number of specimens from the region and based on the geologic availability of the islands (<40 Ma)^19–21^.

In BioGeoBEARS, we used a time slice model based on the breakup of the continents, including the split between Africa and South America, following descriptions in Seton et al.^22^ of the historical geology and modeled after the dispersal models used in Toussaint et al. ^23^. Dispersal rate scalers were used to down-weight the baseline dispersal rates based on the proximity of continents^24^. We used four time slices in the dispersal matrix to reflect the geologic changes from 130 Ma to present. Following Toussaint et al. ^23^, these dispersal rate scalers included (1); small water barrier (0.75); land barrier (0.5); large water barrier (0.25); not yet existent (0.01). The complete dispersal rate matrices for each time slice can be found in Table 4.2.

BioGeoBEARS can implement the(*+j*) parameter, which accounts for jump dispersal or founder event speciation^25^. We compared the weighed ​​Akaike informative criterion wAIC scores of six models: dispersal-extinction cladogenesis (DEC), dispersal vicariance analysis (DIVA), Bayesian analysis of biogeography (BAYAREA), and Bayesian analysis of area-like (BAYAREALIKE). The +*j* parameter has been rejected by Ree and Sanmartín ^24^ because it does not model cladogenetic events when maximized thus rendering non-jump dispersal events impossible. However, because the divergence times are incongruent with historical geologic events, due to the likely importance of jump dispersals we included the +j parameter, even though its current implementation is limited or even dubious.

References

1. Maddison, D. R. & Maddison, W. P. Chromaseq: a Mesquite package for analyzing sequence chromatograms. (2021).

2. Maddison, W. P. & Maddison, D. R. Mesquite: a modular system for evolutionary analysis. (2021).

3. Green, P. Phrap. (2009).

4. Green, P. & Weing, B. Phred. (2007).

5. Faircloth, B. C. Identifying conserved genomic elements and designing universal bait sets to enrich them. *Methods Ecol. Evol.* **8**, 1103–1112 (2017).

6. Kulkarni, S., Wood, H., Lloyd, M. & Hormiga, G. Spider‐specific probe set for ultraconserved elements offers new perspectives on the evolutionary history of spiders (Arachnida, Araneae). *Mol. Ecol. Resour.* **20**, 185–203 (2020).

7. Faircloth, B. C. illumiprocessor: a trimmomatic wrapper for parallel adapter and quality trimming. (2013) doi:10.6079/J9ILL.

8. Bolger, A. M., Lohse, M. & Usadel, B. Trimmomatic: a flexible trimmer for Illumina sequence data. *Bioinformatics* **30**, 2114–2120 (2014).

9. Faircloth, B. C. PHYLUCE is a software package for the analysis of conserved genomic loci. *Bioinformatics* **32**, 786–788 (2016).

10. Prjibelski, A., Antipov, D., Meleshko, D., Lapidus, A. & Korobeynikov, A. Using SPAdes De Novo Assembler. *Curr. Protoc. Bioinforma.* **70**, (2020).

11. Maddison, W. P. *et al.* A phylogenetic and taxonomic review of baviine jumping spiders (Araneae, Salticidae, Baviini). *ZooKeys* **1004**, 27–97 (2020).

12. Starrett, J. *et al.* High phylogenetic utility of an ultraconserved element probe set designed for Arachnida. *Mol. Ecol. Resour.* **17**, 812–823 (2017).

13. Katoh, K. & Standley, D. M. MAFFT Multiple Sequence Alignment Software Version 7: Improvements in Performance and Usability. *Mol. Biol. Evol.* **30**, 772–780 (2013).

14. Capella-Gutierrez, S., Silla-Martinez, J. M. & Gabaldon, T. trimAl: a tool for automated alignment trimming in large-scale phylogenetic analyses. *Bioinformatics* **25**, 1972–1973 (2009).

15. Matzke, N. J. BioGeoBEARS: BioGeography with Bayesian (and Likelihood) Evolutionary Analysis in R Scripts. (2013).

16. Rannala, B. & Yang, Z. Inferring Speciation Times under an Episodic Molecular Clock. *Syst. Biol.* **56**, 453–466 (2007).

17. Holt, B. G. *et al.* An Update of Wallace’s Zoogeographic Regions of the World. *Science* **339**, 74–78 (2013).

18. Turk, E., Čandek, K., Kralj‐Fišer, S. & Kuntner, M. Biogeographical history of golden orbweavers: Chronology of a global conquest. *J. Biogeogr.* **47**, 1333–1344 (2020).

19. Iturralde-Vinent, M. A. Meso-Cenozoic Caribbean Paleogeography: Implications for the Historical Biogeography of the Region. *Int. Geol. Rev.* **48**, 791–827 (2006).

20. Iturralde-Vinent, M. A. Aspectos geológicos de la biogeografía de Cuba. *Cienc. Tierra El Espac.* **5**, 85–100 (1982).

21. Iturralde-Vinent, M. A. & MacPhee, R. D. Paleogeography of the Caribbean region: implications for Cenozoic biogeography. *Bull. AMNH* **238**, (1999).

22. Seton, M. *et al.* Global continental and ocean basin reconstructions since 200Ma. *Earth-Sci. Rev.* **113**, 212–270 (2012).

23. Toussaint, E. F. A., Bloom, D. & Short, A. E. Z. Cretaceous West Gondwana vicariance shaped giant water scavenger beetle biogeography. *J. Biogeogr.* **44**, 1952–1965 (2017).

24. Ree, R. H. & Sanmartín, I. Conceptual and statistical problems with the DEC +J model of founder‐event speciation and its comparison with DEC via model selection. *J. Biogeogr.* **45**, 741–749 (2018).

25. Matzke, N. J. Model Selection in Historical Biogeography Reveals that Founder-Event Speciation Is a Crucial Process in Island Clades. *Syst. Biol.* **63**, 951–970 (2014).
